# The Role of Galectin-1 and Galectin-3 in the Mucosal Immune Response to *Citrobacter rodentium* Infection

**DOI:** 10.1371/journal.pone.0107933

**Published:** 2014-09-22

**Authors:** Renata Curciarello, Alison Steele, Dianne Cooper, Thomas T. MacDonald, Laurens Kruidenier, Takahiro Kudo

**Affiliations:** 1 Centre for Immunology and Infectious Disease, Blizard Institute, Barts and the London School of Medicine and Dentistry, Queen Mary University of London, London, United Kingdom; 2 William Harvey Research Institute, Barts and the London School of Medicine and Dentistry, Queen Mary University of London, London, United Kingdom; Federal University of São Paulo, Brazil

## Abstract

Despite their abundance at gastrointestinal sites, little is known about the role of galectins in gut immune responses. We have therefore investigated the *Citrobacter rodentium* model of colonic infection and inflammation in Galectin-1 or Galectin-3 null mice. Gal-3 null mice showed a slight delay in colonisation after inoculation with *C. rodentium* and a slight delay in resolution of infection, associated with delayed T cell, macrophage and dendritic cell infiltration into the gut mucosa. However, Gal-1 null mice also demonstrated reduced T cell and macrophage responses to infection. Despite the reduced T cell and macrophage response in Gal-1 null mice, there was no effect on *C. rodentium* infection kinetics and pathology. Overall, Gal-1 and Gal-3 play only a minor role in immunity to a gut bacterial pathogen.

## Introduction

Galectins are a family of 15 lectins, each containing a carbohydrate recognition domain with affinity for β-galactoside-containing oligosaccharides. Galectins show considerable functional diversity and can alter immune cell function by acting as both intracellular modulators and secreted effectors [1], [2].

Galectin-3 (Gal-3) is highly expressed in the mucosal epithelium and has pro-inflammatory functions, including immune cell recruitment to, and maintenance at, inflammatory sites [3]–[5]. Gal-3 is a positive regulator of T cell proliferation, it can protect T cells from apoptosis and can activate macrophage and neutrophil phagocytosis and reactive oxygen species production [1], [6]–[10]. In addition, Lippert *et al.* found Gal-3 to be a potent stimulator of colonic lamina propria fibroblasts, causing NF-κB activation and IL-8 production [5].

Galectin-1 (Gal-1) is also highly expressed by colonic epithelial cells but contrary to Gal-3 [11] it has mainly anti-inflammatory functions, including the enhancement of regulatory T cell activity and the inhibition of inflammatory cell recruitment to peripheral sites [12]–[14]. *In vivo* studies support an anti-inflammatory role for Gal-1, and Santucci *et. al.* showed that prophylactic or therapeutic administration of Gal-1 can significantly reduce TNBS-induced colitis by preventing T cell activation and pro-inflammatory cytokine production[11]. Thus, Galectin-1 and Galectin-3 appear to have opposing roles that may act in concert to regulate inflammation and maintain immune homeostasis.

The *in vivo* role of Gal-1 and Gal-3 in mucosal immune responses to bacterial infection has not been previously investigated. *Helicobacter pylori* challenged human gastric epithelial cells show increased expression of both Gal-1 and Gal-3 *in vitro* [15], [16], while in a mouse model of lung infection by *Streptoccus pneumoniae* Gal-3 was found to be increased in infected lungs, where it mediates neutrophil adhesion to endothelial cells and neutrophil recruitment into tissues [17], [18].

To determine the importance of Gal-1 and Gal-3 in immune-mediated pathology and immunity to bacterial infection, we infected Gal-1 or Gal-3 null mice with the non-invasive bacterial pathogen *Citrobacter rodentium*. Infection of wild-type mice with *C. rodentium* results in colitis and a Th1 biased mucosal inflammatory response in the distal colon, providing a useful model of mucosal immune responses [19].

## Materials and Methods

### Ethics statements

Experiments were performed in accordance with the Guidance on the Operation of Animals, Scientific Procedures Act 1986, and were approved by UK Home Office (Licence reference number: PPL 70/6833). Queen Mary University of London is also a signatory to the ARRIVE guidelines (*Animal Research*: *Report In Vivo Experiments*) in the UK which seeks to provide the key information on how an animal study was designed, conducted and analysed. Anesthetized mice (intraperitoneal injection of 100 mg/kg ketamine and 5 mg/kg xylacine) were humanely euthanised by cervical dislocation by experienced research personnel. All efforts were made to alleviate suffering during the whole experiment.

### Mice

Female 6–8 week old Gal-1 or Gal-3 deficient mice (*Lgals1^−/−^* and *Lgals3^−/−^*, respectively) on a C57BL/6 background were kindly provided by Prof. M. Perretti (William Harvey Research Institute, Barts and The London, UK) from breeding colonies held at B&K Universal Ltd (Hull, UK). Control C57BL/6 mice were purchased from Charles River (Kent, UK). Animals were housed in high efficiency particulate air filtered cages with sterile bedding and free access to commercial food and water. Independent infection experiments were performed at least twice using 3 to 6 mice per group. Animals were monitored daily. The license under which these experiments were carried out allows animals to be kept alive until they lose 10% of their body weight after which they must be culled by a schedule 1 procedure. In this case, animals did not lose weight during infection.

### Bacteria and infections

**Figure 1:**
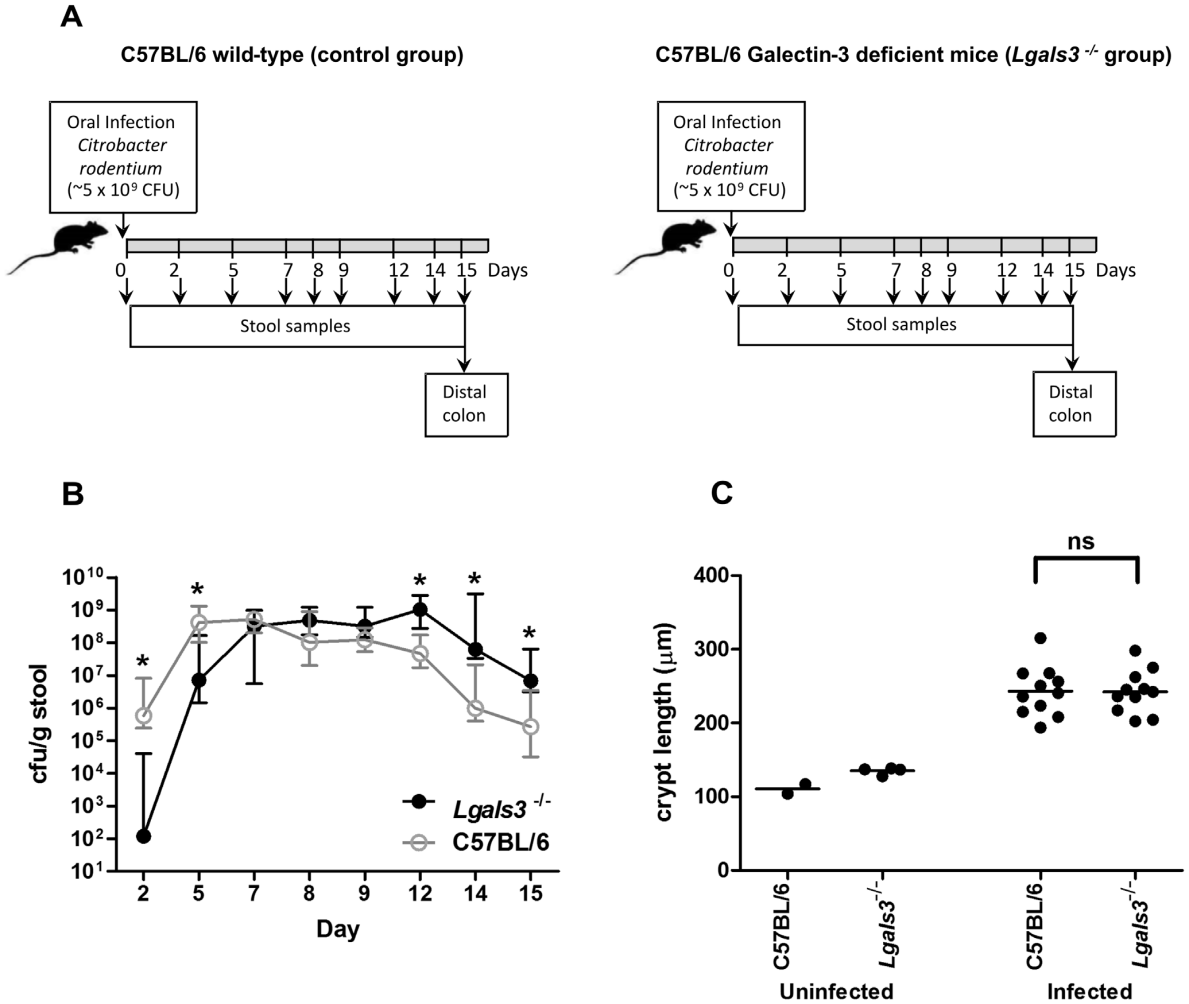
Galectin-3 deficient mice showed delayed colonisation and clearance, and normal crypt hyperplasia after *C.rodentium* infection. **A.** Scheme of the protocol used for infection and sampling; **B.** The colony forming units (cfu) of *C. rodentium* shed per gram of stool by infected C57BL/6 wild-type controls (open circles) and infected Galectin-3 knockout (*Lgals3* ^−/−^) mice (closed circles) is shown per day after infection. Values indicate the median and interquartile range of groups of 11 animals; C. Crypt length at day 15 post infection. Dots indicate the average crypt length per mouse, while horizontal lines indicate the average per group of mice (n = 2–4 for uninfected mice and 9–11 for infected mice). Data was obtained from 2 independent infection experiments, an asterisk indicates a statistically significant difference (p<0.02) and “ns” means no significant difference (p>0.05) between *Lgals3* ^−/−^ and C57BL/6 wild-type controls.

The bacterial strain used in this study, *Citrobacter rodentium* ICC180, was grown in Luria Bertani (LB) medium with 50 µg/ml kanamycin at 37°C with agitation[20]. Mice were orally inoculated with 200 µl of an overnight culture of bacteria resuspended in 1 ml PBS (∼5×10^9^ CFU) using a gavage needle. The viable count of the inoculum was determined by retrospective plating onto LB agar containing kanamycin. As it is outlined in figure 1A, stool samples were recovered aseptically at various time points after inoculation and the number of viable bacteria per gram of stool was determined after homogenization at 0.1 g/ml in PBS and plating onto LB agar containing kanamycin. At day 15 post infection mice were sacrificed and pieces of distal colon were snap frozen in liquid nitrogen before storage at −70°C prior to analysis.

### Hematoxylin and eosin staining of colonic sections

Frozen colonic tissues embedded in OCT mounting medium (Agar Scientific Ltd., Stanstead, UK) were sectioned using a cryostat to a thickness of 5 µm. Sections were mounted on polylysine-coated slides (VWR BDH, Lutterworth, UK), they were air dried overnight and then fixed in acetone for 20 min at room temperature, before drying for a minimum of 1 hour. Sections were then stained according to standard protocols for hematoxylin and eosin staining. The colonic crypt length (as an indication of hyperplasia) was measured using a graticule. Only well-oriented crypts were measured and a minimum of 10 measurements were taken for each sample.

### Immunohistochemistry

Cryostat sections of acetone fixed mouse distal colon were rehydrated in Tris-buffered saline (TBS) for 5 min and then incubated for 1 hour with antibodies against CD3, CD4, CD8, F4/80, and CD11c (AbD Serotec, Oxford, UK). Sections were gently washed with TBS 3 times for 3 min before addition of biotinylated anti-rat IgG (Abcam plc, Cambridge, UK) or anti-hamster IgG (AbD Serotec, Oxford, UK) with 4% (v/v) normal murine serum for blocking (Sera Laboratories International, Sussex, UK) for 30 min at room temperature. After washing, 0.1% avidin-peroxidase conjugate (Sigma-Aldrich Ltd, Dorset, UK) was added for 30 min before further washing and the addition of diaminobenziadine substrate (Sigma-Aldrich Ltd, Dorset, UK) for 5 to 10 min. The reaction was stopped with excess TBS and sections were counterstained with hematoxylin. A control slide using no primary antibody was used to show endogenous peroxidase-containing cells. Stained cell populations were counted in 5 randomly selected fields per section and data were expressed as the number of positive cells per 250 µm^2^ of colonic lamina propria.

### Enzyme linked immunosorbent assay (ELISA)

Proteins were extracted from approximately 15 mg of snap frozen colonic tissue by rapid rotor-stator homogenisation in PBS plus protease inhibitor cocktail (SIGMA- Aldrich, St. Louis, MO, USA), it was sonicated 3 times in 10 second bursts followed by incubation in 1% Igepal lysis buffer for 20 min on ice with occasional vortexing. Supernatants were analysed for cytokine levels using a MesoScale Discovery mouse pro-inflammatory 7-plex ELISA kit (Gaithersburg, MA, USA) and an SI6000 electro-chemiluminescence plate reader, according to the manufacturer's instructions.

### Migration assay

The murine monocytic cell line J774 [21], kindly provided by Dr. Olivier Marches (Barts and The London School of Medicine and Dentistry, London, UK), was maintained in DMEM supplemented with 10% foetal bovine serum, 1% penicillin-streptomycin and 25 µg/ml gentamycin at 37°C and 5% CO_2_ (Sigma-Aldrich Ltd., Dorset, UK). For migration assays, a 24 well transwell plate containing 8.0 µm pore membrane inserts (Appleton Woods, Birmingham, UK) was used. Top wells contained 5×10^4^ J774 cells in 100 µl serum-free DMEM while bottom wells contained 600 µl serum free DMEM plus 100 ng/ml or 1 µg/ml recombinant mouse Gal-1 (R&D Systems, Abingdon, UK). Recombinant mouse monocyte chemoattractant protein-1 (MCP-1) (R&D Systems, Abingdon, UK) at 100 ng/ml was used as a positive control and media alone as a negative control. Migration was allowed to proceed at 37°C and 5% CO_2_ for 6 to 24 hours after which adherent non-migratory cells were removed from the insert with two PBS washes followed by gentle wiping with a sterile cotton bud dipped in PBS. Migratory cells adhering to the underside of the insert were fixed and stained using the Hemacolor Rapid staining set (VWR BDH, Lutterworth, UK) according to the manufacturer's instructions. Cells were counted by 2 independent researchers.

### Statistics

Statistical analysis was carried out using GraphPad Prism 4 software. Data from experiments involving mice and their tissues were analysed by means of a two-tailed Mann Whitney test for non-parametric data, while the migration assay data were analysed by ANOVA with Tukeys post test. A P value of <0.05 was taken as significant in all cases.

## Results

In order to investigate the importance of Gal-1 and Gal-3 in mucosal immune responses to infection, *Lgals1* ^−/−^ and *Lgals3* ^−/−^ mice were infected with the enteric pathogen *Citrobacter rodentium*. Bacterial colonisation levels during infection were monitored by stool sampling, and mucosal immune responses to infection were evaluated through quantification of immune cell influxes into the lamina propria, measurement of the extent of hyperplasia and quantification of cytokine and chemokine levels in the distal colon. The immune responses of *Lgals1^−/−^* and *Lgals3^−/−^* mice to infection are presented separately for clarity.

### Gal-3 deficient mice showed altered colonisation to *C. rodentium* infection

Gal-3 null mice and wild-type controls (C57BL/6) were orally inoculated with *C. rodentium* and the number of bacteria shed per gram of stool was used to monitor mucosal colonisation (Figure 1A). Wild-type mice showed typical colonisation kinetics with infection peaking at approximately 5×10^8^ cfu/g stool between days 5 and 9 post inoculation (p.i.), followed by reducing numbers from day 12 onwards as the bacteria are cleared from the gastrointestinal tract. The *Lgals3^−/−^* mice demonstrated a decreased early colonisation at day 2 and day 5 p.i. (Figure 1B). Although infection peaked at around the same level as wild-type mice from day 7 to 12 p.i., Gal-3 deficient mice shed significantly more bacteria between days 12 and 15, indicating a slightly altered kinetics of infection (Figure 1B). All *Lgals3^−/−^* mice showed a logarithmic decrease in stool shedding at day 15, showing resolution of infection. Although the pathogen burden at day 15 p.i. was significantly greater in Gal-3 null mice, the colonic mucosal hyperplasia characteristic of *C. rodentium* infection was not significantly different to that of wild-type (Figure 1C).

### Lgals3^−/−^ mice showed reduced immune cell infiltration into colonic tissue during *C. rodentium* infection

**Figure 2:**
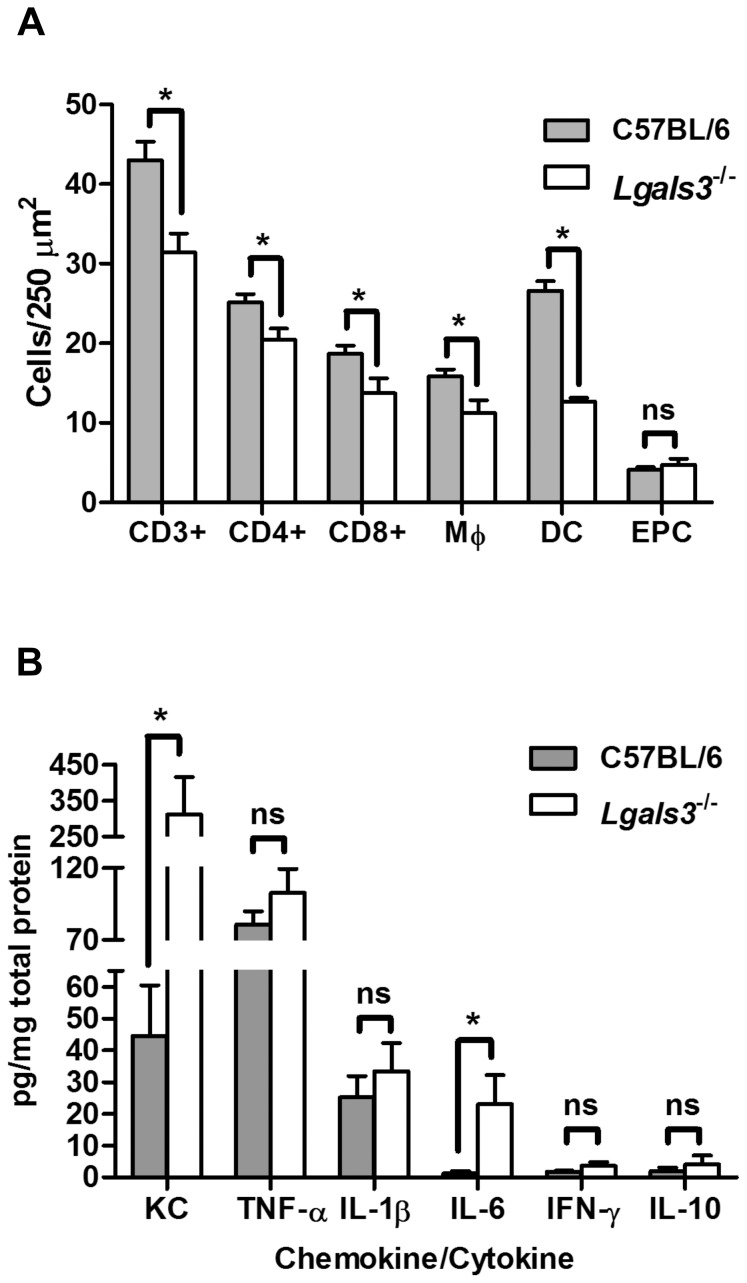
Galectin-3 deficient mice showed reduced inflammatory infiltrates into lamina propria and enhanced expression of IL-6 and KC. **A.** Number of CD3^+^ cells, CD4^+^ cells, CD8^+^ cells, macrophages (M_Φ_), dendritic cells (DC) and endogenous peroxidase-containing (EPC) cells or neutrophils per 250 µm^2^ of colonic lamina propria in *C. rodentium* infected mice. Bars indicate the average for each group of 10 C57BL/6 wild-type and *Lgals3* ^−/−^ animals; **B.** Data show cytokine and chemokines levels as pg per mg of total proteins in the colonic tissue of *Lgals3* ^−/−^ and C57BL/6 wild-type control mice. Data fit Gaussian distribution, bars represent the mean cytokine expression for groups of 10 mice while error bars indicate the SEM. Data was obtained from 2 independent infection experiments. An asterisk indicates a statistically significant difference (p≤0.05) and “ns” means no significant difference (p>0.05) between groups of galectin deficient and control mice.

Since Gal-3 is known to promote the migration and survival of T cells, dendritic cells (DC), macrophages and neutrophils [4], [10], [18], [22], [23], a reduction in inflammatory responses might explain the observed slight delay in bacterial clearance in the Gal-3 deficient mice. *C. rodentium* infection typically elicits an influx of CD4^+^ and CD8^+^ T cells, DC, macrophages and neutrophils [19], [22]. Uninfected *Lgals3^−/−^* and wild type mice showed similar immune cell counts in the lamina propria (data not shown). Infection with *C. rodentium* induced a significant increase in the number of lamina propria CD3^+^ T cells, CD4^+^ T cells, CD8^+^ T cells, DC and macrophages at day 15 p.i. in wild-type mice. However, in agreement with the pro-inflammatory functions of Gal-3, *Lgals3^−/−^* mice showed a significantly reduced immune cell influx compared to *C. rodentium* infected wild-type controls (Figure 2A). There was no however effect on the influx of endogenous peroxidase-containing (EPC) cells (most commonly neutrophils) into the lamina propria (Figure 2A).

### *C. rodentium* infected Lgals3^−/−^ mice showed increased expression of IL-6

**Figure 3:**
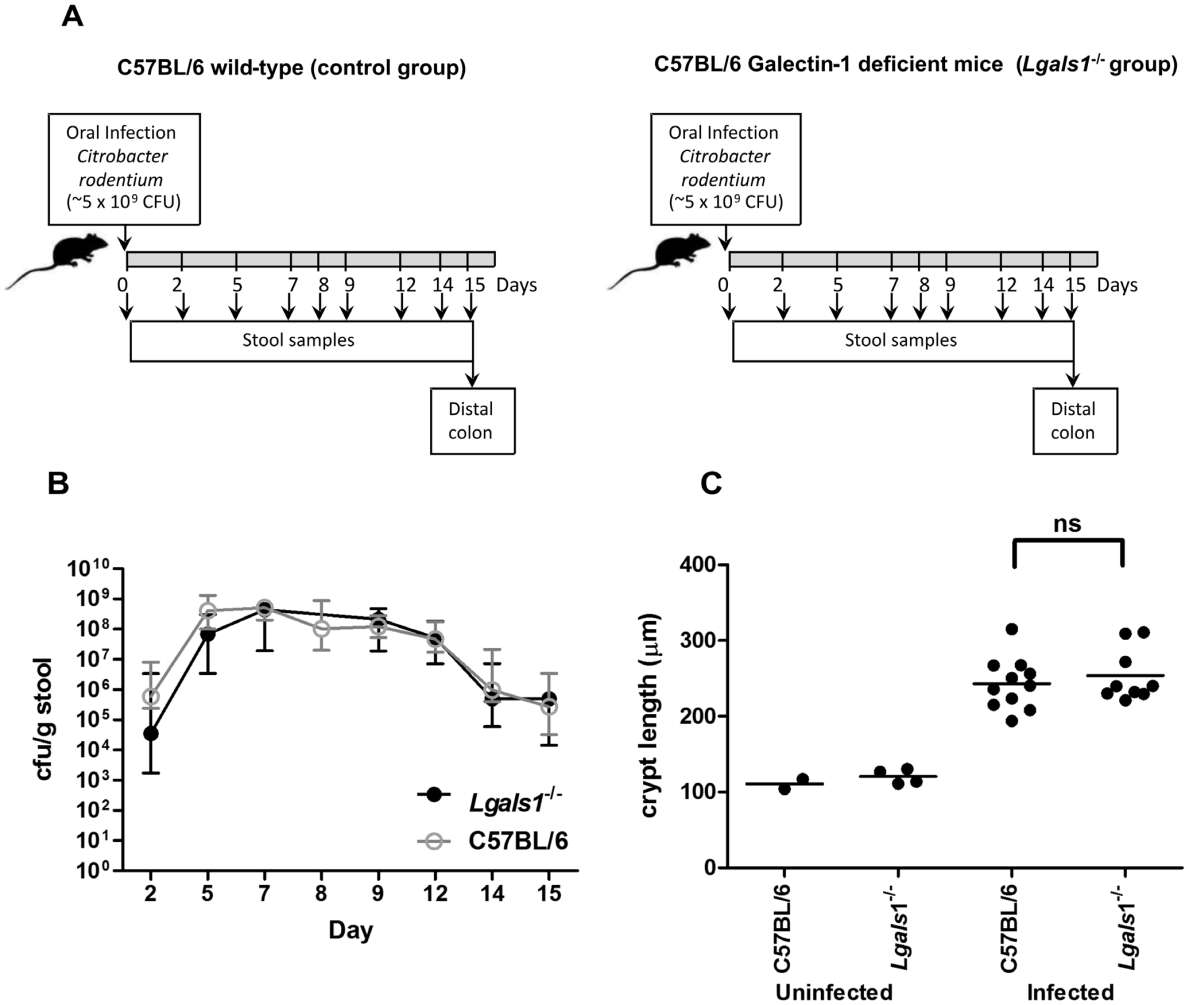
Galectin-1 deficient mice showed infection kinetics similar to wild-type controls and normal hyperplasia levels. **A.** Scheme of the protocol used for infection and sampling; **B.** Data represent colony forming units (cfu) of *C. rodentium* shed per gram of stool by infected C57BL/6 wild-type controls (open circles) and infected Galectin-1 knockout (*Lgals1* ^−/−^) mice (closed circles) at different stages of the infection plan. Dots indicate the median and interquartile range of groups of 10 animals; **C.** Crypt length at day 15 post infection is plotted. Dots indicate the average crypt length per mouse, while horizontal lines indicate the average per group of mice (n = 2–4 for uninfected mice and 9–11 for infected mice). Data was obtained from 2 independent infection experiments, “ns” means no significant difference (p>0.05) between infected galectin deficient and wild-type mice.

We next quantified the pro-inflammatory cytokines interferon gamma (IFN-γ), tumor necrosis factor alpha (TNF-α), interleukin (IL)-1β, and IL-6, and the anti-inflammatory cytokine IL-10, in infected colonic tissue by ELISA. The neutrophil chemoattractant keratinocyte-derived chemokine (KC) was also quantified. Control mice showed undetectable levels of these proteins in the gastrointestinal tract prior to infection (data not shown). *C. rodentium* infected *Lgals3^−/−^* mice showed TNF-α, IFN-γ and IL-1β levels similar to wild-type controls at day 15 p.i. (Figure 2B). IL-10 was present at low levels and was not significantly different between *Lgals3^−/−^* mice and wild-type controls (Figure 2B). Interestingly IL-6, which has been implicated in protection of the epithelium during *C. rodentium* infection [23], was significantly increased in mutant mice compared with wild-type controls at day 15 p.i. (Figure 2B). KC was also significantly increased compared to infected wild-type controls (Figure 2B) but, as shown in figure 3A, there was no increase in the number of neutrophils in the lamina propria at day 15 p.i.

### Galectin-1 deficiency has no effect on *C. rodentium* infection kinetics or hyperplasia

Next we investigated the effect of deficiency in the anti-inflammatory lectin Gal-1 in *Lgals1^−/−^* mice infected with *C. rodentium* (Figure 3A). These mice demonstrated colonisation kinetics that closely followed wild-type infection (Figure 3B), suggesting that Gal-1 does not play a role in immunity to *C. rodentium* infection. Uninfected *Lgals1^−/−^* mice exhibited normal crypt architecture (Figure 3C) and the colonic mucosal hyperplasia induced by *C. rodentium* infection was not significantly different from that of wild-type at day 15 p.i. (Figure 3C).

### Lgals1^−/−^ mice showed reduced T cell and enhanced neutrophil infiltrates into colonic tissue during *C. rodentium* infection

**Figure 4:**
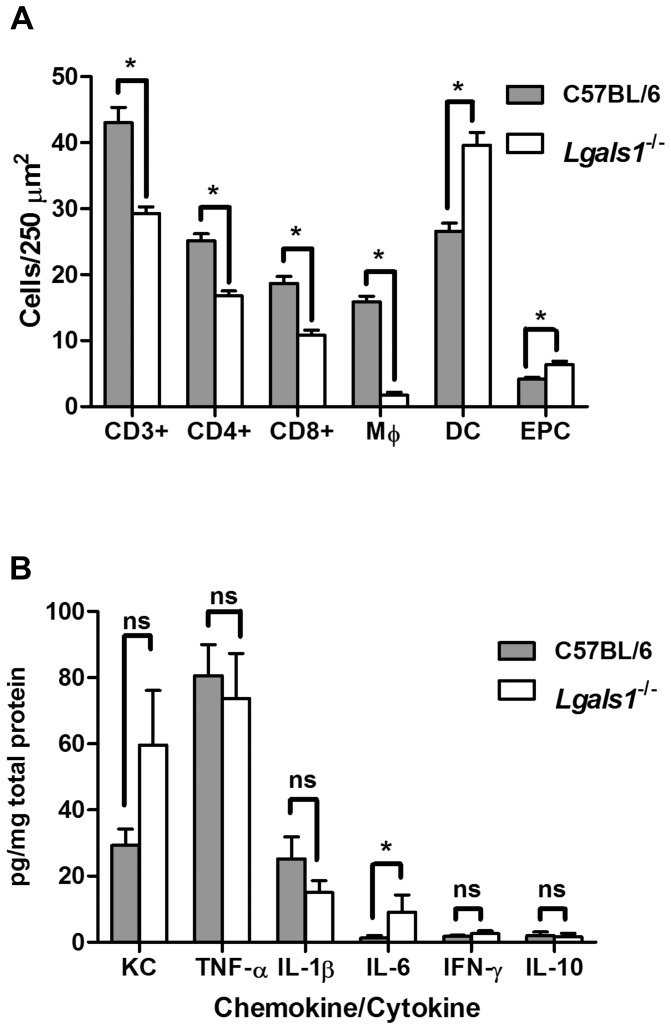
Galectin-1 deficient mice showed increased neutrophil and DC infiltrates and enhanced expression of IL-6 and KC. **A.** Number of CD3^+^ cells, CD4^+^ cells, CD8^+^ cells, macrophages (M_Φ_), endogenous peroxidase-containing (EPC) cells (most commonly neutrophils) and DC (dendritic cells) per 250 µm^2^ of colonic lamina propria in *C. rodentium* infected mice. Bars indicate the average for each group of 10 C57BL/6 wild-type and *Lgals1^−/−^* animals; **B.** Data show cytokines and chemokine levels as pg per mg of total proteins in the colonic tissue of *Lgals1^−/−^* and C57BL/6 wild-type control mice. Data fit Gaussian distribution, bars represent the mean expression for groups of 10 mice while error bars indicate the SEM. Data was obtained from 2 independent infection experiments, an asterisk indicates a statistically significant difference (p≤0.05) and “ns” means no significant difference (p>0.05) between galectin and wild-type control mice.

T cell infiltrates in the lamina propria of *Lgals1^−/−^* mice were investigated by immunohistochemistry. On infection with *C. rodentium*, *Lgals1^−/−^* mice showed a significant decrease in the number of CD3^+^, CD4^+^ and CD8^+^ T cells compared to uninfected control mice at day 15 p.i. (Figure 4A). Conversely, *Lgals1^−/−^* mice exhibited greater number of EPC cells in the colonic lamina propria at day 15 p.i. than wild-type controls (Figure 4A). The reduced lamina propria T cell infiltrate of *C. rodentium* infected *Lgals1^−/−^* mice (Figure 4A) was similar to that observed in infected *Lgals3^−/−^* mice (Figure 2A).

### *C. rodentium* infected Lgals1^−/−^ mice showed increased expression of IL-6 and KC similar to Lgals3^−/−^ mice

Cytokines were quantified in the colonic tissue of *C. rodentium* infected *Lgals1^−/−^* mice and we found that the content of TNF-α, IFN-γ, IL-1β and IL-10 were statistically similar to infected wild-type control animals (Figure 4B). Interestingly, the pro-inflammatory cytokine IL-6 and the neutrophil chemoattractant KC were increased in *Lgals1^−/−^* mice, as it was observed in *Lgals3^−/−^* mice (Figure 2B). Although the increased level of KC in *Lgals1^−/−^* mice was no statistically significant (Figure 4A), the trend may be consistent with significant increase of EPC cells in the colon of infected *Lgals1^−/−^ mice* (Figure 4B).

### Galectin-1 induced the migration of monocytes *in vitro*

**Figure 5:**
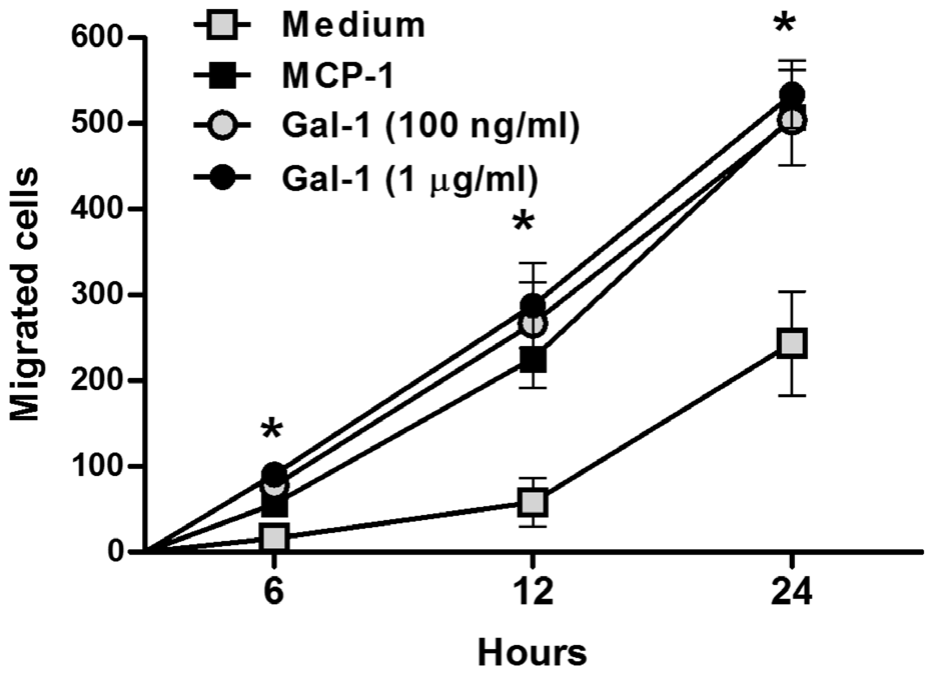
Galectin-1 induces migration of J774 monocytes *in vitro*. Number of J774 monocytes that cross a transwell membrane when stimulated with 100 ng/ml monocyte chemoattractant protein (MCP-1, black squares), 100 ng/ml Gal-1 (grey circles) or 1 µg/ml Gal-1 (black circles) for 6, 12 and 24 hours. Each symbol represents the mean and SEM from 3 experiments in which 5 counts per membrane were taken by 2 independent researchers. An asterisk indicates a statistically significant difference (p<0.05) between Gal-1 treated wells and media only controls.

Since *Lgals1^−/−^* mice had significantly fewer lamina propria resident macrophages than wild-type mice, and these mice did not demonstrate a macrophage response to infection with *C. rodentium*, we hypothesised that Gal-1 might have chemoattractant function for the monocytic precursors of lamina propria macrophages. To test this hypothesis, we used the murine monocytic cell line J774 in a transwell system to quantify the migratory activity of Gal-1 compared to the known MCP-1. We found that Gal-1 significantly induced the migration of monocytes, similarly to MCP-1 (Figure 5), suggesting that Gal-1 might act as a mucosal chemoattractant for monocytes *in vivo*.

## Discussion

We investigated the importance of galectins in immunity to bacterial pathogens at the gastrointestinal surface in a mouse model of infection with *C. rodentium*. Although Gal-3 deficiency has been reported to have no effect on resolution of *Trichuris muris* nematode infection [24], in this work *Lgals3^−/−^* mice demonstrated a significant delay in clearance of the non-invasive bacterial pathogen *C. rodentium* following the oral administration, which is associated with a significant reduction in the recruitment of T cells, DC and macrophages to the lamina propria of the colon, which likely promoted a delayed clearance of this pathogen *in vivo*. Our data therefore supports previous experiments demonstrating that Gal-3 deficient mice have defects in immune cell recruitment and maintenance at inflamed sites and supports the hypothesis that Gal-3 exerts a mucosal pro-inflammatory effect *in vivo* on T cells [3], [4], [25], macrophages and DC [1], [26], [27]. Therefore, for the first time Gal-3 deficiency has been shown to result in delayed clearance of a pathogen. Although *Toxoplasma gondii* infection of Gal-3 null mice resulted in increased pathogen burdens in the lungs and brain and reduced leukocyte recruitment, the infection was resolved normally, probably due to enhanced Th1 responses with heightened IFN-γ expression and a high serum IgG2a:IgG1 ratio [3]. In another study, Gal-3 null mice infected with *Schistosoma mansoni* also demonstrated reduced immune cell recruitment and a Th1-biased immune response, which resulted in reduced liver granuloma formation. However, no differences in parasite burden nor resolution of infection was observed between deficient and wild-type mice [25]. In addition, we observed that *Lgals3^−/−^* mice showed a considerable delay in intestinal colonisation after oral challenge with *C. rodentium*. Since Gal-3 is located at the apical surface of confluent colonic T84 cells [28], it is exclusively secreted from the apical surface of polarised kidney epithelial cells [29], it is produced by colonic epithelial cells closely located to the luminal surface and it is attached to mucin [30], [31], we propose that Gal-3 might constitute a binding target for *C. rodentium* and a deficiency in this lectin might negatively affect the ability of *C. rodentium* to colonize the intestinal mucosa. Other works have demonstrated that Gal-3 aggregates are coating indigenous bacteria in the gastrointestinal tract of mice and Gal-3 can bind directly to β-galactoside-containing membrane lipopolysaccharide (LPS) from *Klebsiella pneumoniae* [32], *Salmonella Minnesota*, *Salmonella typhimurium*, *Neisseria gonorrhoeae* and *Helicobacter pylori* [15], [33], [34]. Indeed, over expression of Gal-3 in gastric epithelial cells results in enhanced adhesion of *H. pylori* to cells *in vitro* [15]. Preliminary data from confocal microscopy and ELISA experiments indicate that *C. rodentium* might express a Gal-3-like surface protein as well as Gal-3 receptors (unpublished data). However, additional components may be involved in the colonisation of this bacteria to the mucosa surface since similar colonic burdens were reached in Gal-3 deficient mice and wild-type controls, and the infection process was not shortened by a deficiency in Gal-3. Likewise, demonstrated that although *H. pylori* could bind to Gal-3, this binding was not the only adherence mechanism employed by the pathogen during colonisation of gastric epithelial cells *in vitro* [15].

Contrary to previous works demonstrating reduced neutrophil recruitment in Gal-3 deficient mice in the lung mucosa during streptococcal infection and into the peritoneal cavity during *T. gondii* challenge [3], [17], we did not observe a reduction in lamina propria neutrophil recruitment during *C. rodentium* infection. Instead, *Lgals3^−/−^* mice demonstrated KC expression 10 times higher than infected controls. This data might suggest that Gal-3 deficiency might affect the ability of neutrophils to respond to the KC chemokine, possibly due to reduced neutrophil adhesion to endothelial cells and ECM proteins, functions normally enhanced by the actions of Gal-3 [18], [35].

Regarding Gal-1, although this lectin is a cervical epithelial cell receptor for the sexually transmitted parasite *Trichomonas vaginalis* and can promote HIV-1 absorption to CD4^+^ T cells and macrophages facilitating the infection [36], [37], in this work we observed no effect on *C. rodentium* colonisation of the mucosal epithelium.

Unexpectedly, we found that *Lgals1* ^−/−^ mice had diminished T cells and macrophages in the colonic lamina propria during infection with *C. rodentium* as compared to wild type mice and similarly to *Lgals3* ^−/−^ mice. Therefore, the deficiency in Gal-1 or Gal-3 resulted in a similar reduction in the T cell influx into the lamina propria on infection, which might suggest these lectins have a similar but non-redundant function with regards to T cell responses. A reduction in macrophage and DC antigen presentation might account for the reduced T cell responses in *Lgals3* ^−/−^ mice, whereas profound macrophage deficiency or the loss of a macrophage derived factor might account for the T cell responses observed in *Lgals1* ^−/−^ mice. Interestingly, Gal-1 deficient mice had very few lamina propria macrophages prior to infection, and failed to mount a macrophage response to infection, at least at the time point considered. This prompted us to hypothesise that Gal-1 might play a role in the recruitment of the monocytic precursors of macrophages to the colonic epithelium. Indeed, the transwell assays led us demonstrate that Gal-1 induced migration of the monocytic cell line J774 *in vitro*. This data is supported by the work of Malik et.al [38]. Despite evidences that monocytes can differentiate into both DC and macrophage [39], [40], we found higher amount of DC in the lamina propria of Gal-1 deficient mice, which might suggest that monocyte recruitment is not affected by Gal-1 deficiency.

The enhanced recruitment of DC and neutrophils in the lamina propria might be responsible for the normal resolution of infection. These data support the hypothesis that Gal-1 has anti-inflammatory functions *in vivo* and might act to limit the recruitment of DC and neutrophils to sites of inflammation. Indeed, previous work has demonstrated that Gal-1 can directly inhibit DC migration to draining lymph nodes *in vivo*, it can inhibit neutrophil adhesion and migration and enhances neutrophil phagocytosis by macrophages [41]–[45].

The loss of these Gal-1 mediated inhibitory functions might explain the enhanced DC and neutrophil responses observed in *C. rodentium* infected Gal-1 deficient mice.

Although our *in vivo* data supports some of the proposed pro-inflammatory functions of Gal-3 and anti-inflammatory functions of Gal-1, we found surprising similarities in the responses of *Lgals3* ^−/−^ and *Lgals1* ^−/−^ mice to *C. rodentium* infection. Hyperplasia was normal in both groups of mice, perhaps as a result of heightened IL-6 production which acts to protect the epithelium from apoptosis during *C. rodentium* infection to maintain barrier function [23]. Both deficient mice showed similarly reduced CD3^+^ T cell responses, reduced macrophage responses, unchanged IL-10, IL-1β, TNF-α and IFN-γ cytokine responses and increased KC chemokine expression, although *Lgals3* ^−/−^ mice showed significantly more expression of these cytokines and chemokines than *Lgals1* ^−/−^ mice. Taken together, these data might suggest that Gal-1 and Gal-3 are not as opposed in function as previously hypothesised.

In conclusion, we have demonstrated that Gal-3 plays an important role in the mucosal immune response to infection with the bacterial pathogen *C. rodentium* and might constitute a binding partner for *C. rodentium in vivo*. Gal-1 also plays a role in gastrointestinal immune responses to bacterial infection, particularly with regard to T cell and macrophage responses, but can be compensated to maintain normal colonisation and infection resolution.
